# Contribution to Family, Friends, School, and Community Is Associated With Fewer Depression Symptoms in Adolescents - Mediated by Self-Regulation and Academic Performance

**DOI:** 10.3389/fpsyg.2020.615249

**Published:** 2021-01-20

**Authors:** Ana Kurtovic, Gabrijela Vrdoljak, Marina Hirnstein

**Affiliations:** ^1^Department of Psychology, Faculty of Humanities and Social Sciences, J. J. Strossmayer University of Osijek, Osijek, Croatia; ^2^Department of Psychosocial Science, Faculty of Psychology, University of Bergen, Bergen, Norway

**Keywords:** positive youth development (PYD), youth, gender, self-regulation, academic performance, contribution, community, five Cs model

## Abstract

The tendency to get involved in helping one’s family, friends, school, and community has many potential benefits such as greater compassion, concern for others, and social responsibility. Research interest in the benefits of contribution in adolescents has increased recently, but there are not many studies examining the effect of contribution on adolescents’ mental health. The present study focused on whether the contribution is associated with fewer self-rated depression symptoms in adolescents. We further tested whether self-regulation and academic performance can have a mediating role in this association. A total of 423 secondary school students (233 female) from eastern Croatia participated in the study. Mean age was 16.78 (*SD* = 1.21). Students completed measures of self-regulation, depression symptoms, and contribution (helping one’s family, friends, or neighbors, mentoring peers, volunteering in one’s community, and participating in school organizations or boards), and gave information about age, gender, and academic performance. A hierarchical regression analysis revealed that contribution, self-regulation, and academic performance were related with lower levels of self-rated depression symptoms. Furthermore, mediation analysis indicated a significant indirect effect through two mediators, self-regulation and academic performance, which was stronger than a path containing only self-regulation. Academic performance alone was not a significant mediator. Our findings suggest that contribution could protect against depression by promoting self-regulation, leading to higher academic performance, and consequently fewer depression symptoms.

## Introduction

Research interest in the effects of adolescents’ involvement in their community and society has increased in recent years. Ecological models of adolescent development have long since emphasized the interplay of a person and his or her surroundings in developmental trajectories. However, the engagement of adolescents in contributing to their family, friends, school, and community, whether it is through structured activities such as volunteering or unstructured actions, has not been the focus of many studies until recently. There has been an increase in studies about contribution to family, friends, school, and community (in further text shortened to: contribution) among adolescents and its effects on well-being, partly due to evolvement of positive psychology and positive youth development models, and partly as a response to concerns regarding adolescents being too self-centered and disengaged from their communities (Benson et al., 2006; Smetana et al., 2006; Bowers et al., 2010). Recently more research attention has been given to the effects of volunteering and, especially, activism on well-being once the activist is committed (Montague and Eiroa-Orosa, 2017).

Positive youth development refers to the conception of youth development focused on nurturing youth strengths and potential for positive development, as opposed to the traditional focus on reducing their deficits and weaknesses (Benson et al., 1999; Lerner et al., 2000; Benson, 2007; Lerner, 2017). One of the most influential positive youth development models is the so-called 5Cs plus one model (Lerner et al., 2015). The five Cs here refer to five indicators of positive youth development, namely, confidence, competence, connection, character, and caring or compassion. Contribution was later added as the sixth C of the positive youth development and is seen as a result of the development of the other five indicators (Lerner, 2004, 2009, 2017; Lerner et al., 2005, 2006, 2015). Contribution refers to acts of engaging in civil society and helping one’s family, friends, school, and community, and is an established term in the field of positive youth development (Lerner, 2004, 2009). Its operationalization in this article is based on previously studies investigating the same concept (e.g., Jelicic et al., 2007; Lewin-Bizan et al., 2010; Agans et al., 2014; Conway et al., 2015; Wiium, 2017). The terms contribution, youth contribution, contribution to community, community contribution, youth community contributions, contribution to others, contributions to context, contribution to family, community, and civil society, and contribution to society are sometimes used interchangeably in the literature (e.g., Lerner et al., 2005, 2006; Jelicic et al., 2007; Lewin-Bizan et al., 2010; Conway et al., 2015; Wiium, 2017).

The tendency to get involved in helping one’s family, friends, school, and community, whether it is on a micro-level, such as helping with family finances or projects or helping peers or neighbors, or on a macro-level, such as volunteering in the community, has many potential benefits. Previous studies show that involvement in community service is related to greater compassion (Yates and Youniss, 1996) and concern for others (Hedin and Conrad, 1980; Kirkpatrick Johnson et al., 1998), as well as social responsibility (Flanagan, 2004) and future involvement in civic organizations (Metz and Youniss, 2005).

Some studies demonstrated that volunteering is related to lower levels of depression and better functioning in older adults (Morrow-Howell et al., 2003; Li and Ferraro, 2005; Hong and Morrow-Howell, 2010). While there are studies demonstrating positive effects of adolescents’ involvement in organized activities such as school clubs and performance arts on their well-being (Mahoney, 2000; Mahoney et al., 2002, 2003), there are fewer studies examining the effect of contribution on adolescents’ mental health. Most of the studies that looked at the association between contribution in adolescents and their mental health focused on volunteering or community service within an organized setting and found that it was related to better self-image, self-esteem, and academic achievement, as well as lower depression and other internalizing symptoms, and lower levels of aggression in adolescents (Switzer et al., 1995; Wilson and Musick, 1999; Smetana et al., 2006; Phelps et al., 2007; Bowers et al., 2010). Voluntary activities that include higher personal engagement can provide an individual with a feeling of mastery and empowerment (see Montague and Eiroa-Orosa, 2017, for a review).

There are multiple reasons why contributing to one’s own family, friends, school, and community may be beneficial to adolescents. Adolescence is a period of identity formation (Erikson, 1968). Participating in acts aimed at helping family, friends, school, or a wider community can influence the consolidation of an identity of a helping, caring person. If that happens, it is likely that a person would think of oneself as a good person, which is a basis for higher self-esteem and cognitive consonance and a strong protective factor against mental health problems (Kirkpatrick Johnson et al., 1998). Given the fact that components of one’s self-concept, which develop in adolescence, are likely to be retained throughout adulthood, the forming of an “altruistic” or “helping” identity could protect a person from mental health problems (Hanks and Eckland, 1978; Fischer and Schaffer, 1993). Furthermore, adolescence is a period with many emotional and cognitive changes, one of them being the increase in the capacity for empathy (Call et al., 1995). While this is a normative developmental change, which has positive effects on adolescents’ social functioning, it can also increase distress. Empathizing with problems and emotions of others can be a burden for adolescents who have not yet developed adequate coping mechanisms and self-regulation. Volunteering in the community, helping peers, neighbors, or family members can help adolescents feel less helpless and worried about the problems of others. It can also positively affect self-confidence and self-efficacy because it is a proactive engagement, usually with immediate proof of its effects (Kirkpatrick Johnson et al., 1998). In addition, helping family, friends, school, or one’s community is usually perceived as a positive experience, whether it is simply a good feeling because one was able to help someone, because of positive feedback from peers or adults, or because of meeting new people and having pleasurable interactions with them (Hamilton and Fenzel, 1988). Experiencing positive events and interactions during childhood and adolescence was shown to be very important in preventing mental health problems, especially depression (Zhou and Chen, 2017). Taken together, the findings of studies conducted so far indicate that contribution could be a protective factor against depression.

Furthermore, developmental trajectories are rarely direct especially in developmental psychopathology. Outcomes in children and adolescents are a result of multiple factors, which can affect children and adolescents both directly and indirectly (Cicchetti and Toth, 1998; Masten, 2006; Lewis and Rudolph, 2014). In order to better explore the role of contribution in explaining depressive symptoms in adolescents, our aim was to examine not only possible associations between contribution and depression, but also possible pathways of this association. In order to do that, we focused on self-regulation and academic performance for two reasons: (1) their effects on adolescent depression are well documented (Herman et al., 2008; Kring and Sloan, 2009; Berking et al., 2013; Aldao and Dixon-Gordon, 2014; Huang, 2015; Sloan et al., 2017) and (2) there is a sound theoretical framework for the assumption that self-regulation could be the mechanisms through which contribution affects depression in adolescents.

Self-discrepancy theory (Higgins, 1987) could aid explaining why contribution affects adolescents’ mental health. It posits that people have three kinds of self-representations (actual, ideal, and ought), and that discrepancies between them determine different kinds of unpleasant emotions. Discrepancy between actual and ought self leads to anxiety related emotions, while discrepancy between actual and ideal self leads to depression related emotions. One’s reactions to certain goal-related situations depend on which of these self-guides one is using, and whether one perceives that discrepancies exist. Emotional, behavioral, and cognitive consequences, in terms of trying to reduce the discrepancy, depend largely on one’s ability to self-regulate. Self-regulation is generally described as the flexible regulation of cognition, behavior, and emotion (Bridgett et al., 2015), which includes both unconscious and conscious processes that affect the ability to control responses (Carver, 2004). It is a skill that affects one’s ability to tolerate unfulfilled needs, handle disappointments and failures, and work toward success (Bandy and Moore, 2010).

Developmental systems theories emphasize the plasticity that enables change during human development. Plasticity is initiated from regulation of the relationship between individual and environmental factors (family and community). Healthy and positive development occurs when individual and environmental relations are beneficial to both sides which is referred to as adaptive developmental regulations (Lerner, 2004; Lerner et al., 2005). It is reasonable to assume that behavior involved in contribution to one’s family, friends, school and community leads to less self-discrepancy between actual and ideal self (if a person holds prosocial behavior as one of his or her values), or between actual and ought self (if a person is merely trying to conform to the expectations of others). Given the fact that contribution is a voluntary, intrinsically motivated activity, favorable conclusions about oneself could have positive effects on a person’s ability to regulate behavior and cognition toward minimizing discrepancies between actual and ideal self or ought self across different situations. In other words, self-regulation should be a significant factor for explaining the link between contribution and individual’s depression. Furthermore, Strauman and Eddington (2017) emphasized the importance of self-regulation in psychopathology. They draw on self-discrepancy theory (Higgins, 1987) and regulatory focus theory (Higgins, 1997), according to which pursuing and attaining one’s goals is regulated by approach and promotion system of self-regulation, respectively. They proposed a framework for conceptualizing depression in terms of dysfunctional self-regulation, arguing that a dysfunction in promotion system of self-regulation leads to repeated failures in pursuing and attaining one’s goals. According to the promotion system of self-regulation, not attaining one’s goals leads to depression, because it keeps a person in a vicious cycle of maladaptive cognitions and strategies without the ability to change.

The ability to regulate behavior and emotions at a level appropriate for any given situation, whether with peers and adults or when facing challenges, is highly predictive of relationship success and well-being (Hair et al., 2001; Verzeletti et al., 2016). Adolescents high in self-regulation are less likely to be overwhelmed by potentially stressful situations, which allows them to focus on others or on the task at hand, rather than their own unpleasant emotions (Murphy et al., 1999).

Research also demonstrated positive effects of self-regulation on higher academic achievement, school engagement, peer acceptance, ability to delay gratification, and healthy eating patterns, as well as lower avoidance, less negative behaviors, and depression (Long and Lerner, 1974; Bandy and Moore, 2010; Strauman and Eddington, 2017). Therefore, development of self-regulation benefits different aspects of functioning in adolescents. Studies also showed that self-regulation in children and adolescents can be promoted by cognitive-behavioral training, social skills training, and community service (Bandy and Moore, 2010). Prosocial behaviors (such as community work) have been linked to development of other positive social and emotional outcomes. Specifically, prosocial children are viewed as considerate, good at solving social problems, and less aggressive (Hair et al., 2001). Furthermore, Eisenberg and Fabes (1992) suggested a model that assumes that children who are high in dispositional tendency to perform prosocial acts are well-regulated and constructive in coping, more popular with higher social skills, and low in the dispositional tendency to experience negative emotions, that is, neuroticism. In addition, Edwards et al. (2007) emphasized that positive social development of youth partly depends on opportunities for prosocial behavior available to them. Prosocial behaviors are associated with sympathy (Eisenberg et al., 1997), and sympathy for others has been related to temperament (including attentional control), low negative emotionality in children (Eisenberg, 2000), and regulatory abilities (Hair et al., 2001), all of which promote good interpersonal relations and protect against depression (Beck and Alford, 2009).

Finally, according to a competence-based model of depression (Cole et al., 2001), failure in an important task, especially if repeated, is likely to lead to the development of a negative self-scheme, which increases the risk for depression. Since academic achievement is one of the most important goals in adolescence, and self-regulation determines one’s behavior in terms of goal pursuit, we assumed that academic performance could be an important link between contribution and depression symptoms in adolescents.

Therefore, the purpose of our study was to examine the relation between contribution and depression, as well as possible mediation through self-regulation and academic performance in Croatian adolescents. Most of the studies in this field were done in the United States and there is a need for research in non-American contexts. Although there is significant number of studies examining mental health problems in adolescents in Croatia (e.g., Vulic Prtoric and Macuka, 2006; Kozjak Mikic et al., 2012; Vulic Prtoric, 2016; Kurtovic et al., 2017; Kurtovic, 2018), large scale studies are missing (Novak and Basic, 2008; Markanovic and Jokic-Begic, 2011). The data that do exist are consistent with studies from other countries showing that up to 20% of adolescents in Croatia experience moderate to severe mental health problems, while up to 10% experience depression (Boricevic Marsanic et al., 2017; Ledinski Ficko et al., 2017). However, research in adolescent mental health have mostly focused on risk and protective factors (Vulic Prtoric and Macuka, 2006; Kurtovic et al., 2017; Kurtovic, 2018, 2020). A framework for research and implementation of strategies for positive youth development in a context of mental health promotion in Croatia is yet to be developed (Novak and Petek, 2015; Novak et al., 2019). Specifically, we are not aware of any studies in Croatian context examining the effects of contribution on adolescent mental health. Therefore, the research questions we tried to answer were as follows:

1. Is contribution related to depression symptoms?
2. Is the relationship between contribution and depression symptoms mediated by self-regulation and academic performance?

## Materials and Methods

### Participants and Procedure

The research was conducted within the Positive Youth Development Cross-National Project. A total of 423 secondary school students (233 female and 190 male) participated in the study. In the Croatian education system, primary school lasts 8 years, and children start it at the age of 6 or 7. After primary school, students enroll in secondary schools that last for 3 years (for vocational schools) and 4 years (grammar schools, medical schools, or technical schools). Mean age of participants in this study was 16.78 (*SD* = 1.21). Five secondary schools of different profiles (e.g., gymnasium, medical, technical, and vocational) from eastern Croatia were involved in the study. The study was approved by institutional ethics committees: Ethics Committee of the Faculty of Humanities and Social Sciences in Osijek (class: 602-04/18-01/29, number: 2158-83-02-18-2) and NSD - Norwegian Centre for Research Data (approval number is 51708/3/IJJ). Written permissions from each school headmaster were obtained. Before completing the questionnaire, participants signed informed consent to participate in the survey. Participants were informed about purpose of the study, potential advantages and disadvantages, and what will happen with the information they provided. The participation was anonymous and voluntary, and the participants were made aware that they could terminate their involvement in the study at any time without consequences. The questionnaires were administered on paper during regular classes. The questionnaires were administered either by undergraduate students trained in data collection for this study or one of the coauthors of this study who explained the purpose of the study before distributing the questionnaires. The goal was to get a range of scores in the sample that is as accurate reflection of the population as possible, so all students got an invitation to participate. That includes all students who are able to follow the curriculum of secondary schools. The setting during data collection was equivalent to class setting during regular assessments which provided students with enough distance to answer the questions honestly. Persons who administered the questionnaires did not have access to participants’ identity and did not affect the participants’ decision to participate in the study or provide answers. School staff (head-teachers, teachers, psychologists, or pedagogues) informed the students about the study but were not involved in data collection. Students were made aware that they can refer to their school psychologist, as well as the local mental health and social services in case they had felt distressed during the study. At the beginning of each questionnaire set, there were questions about socio-demographic variables and school grades, followed by scales measuring depression, self-regulation, and contribution.

### Measures

Most parts of the study invitation and information, as well as all items included in the questionnaire were originally written in English and translated to Croatian. In the process of translating scales, we followed the guidelines by Beaton et al. (2000). The original scales were first translated to Croatian independently by two native Croatian speakers. They discussed their translations and reached a consensus with the help of an independent observer. The agreed translated version was then translated back to English by two translators who were not aware of the original English version. All translators and observers had then discussed the translations and agreed on the pre-final version in Croatian, which was then tested on a convenient pilot sample of 30 university students who provided oral feedback on their comprehension and interpretation of the items. After receiving the participants’ feedback, the involved translators agreed on the final Croatian version of the scales.

Contribution (short for: Contribution to family, friends, school, and community) was measured using a scale consisting of five items based on work of Jelicic et al. (2007), Lewin-Bizan et al. (2010), Agans et al. (2014), and Wiium (2017). Participants were asked how many hours per week they spend engaged in different behaviors aimed at helping others or their community. The items covered helping one’s family, friends, or neighbors, mentoring peers, volunteering in one’s community and participating in school organizations or boards (e.g., “*How many hours do you spend in a typical week participating in school committees or government?*”). The answers were offered on a scale from 0 (*0 h per week*) to 5 (*6 h or more per week*). The total score for contribution was calculated by adding together the scores for each item. The total score can range from 0 to 25, with higher scores indicating more contribution. Cronbach’s alpha (*α*) was 0.70.

Self-Regulation Questionnaire (Novak and Clayton, 2001) was used to measure self-regulation. It is a 13-item questionnaire assessing ability to regulate negative emotions and disruptive behavior and to set and attain goals. Respondents rate how true each item is for them, ranging from 1 (*never true*) to 4 (*always true*). The questionnaire can be used as a measure of overall self-regulation or scores can be calculated individually for emotion (e.g., “*I get upset easily*”), cognitive (e.g., “*Once I have a goal, I make a plan to reach it*”), and behavioral (e.g., “*I slam doors when I am mad*”) regulation. We used it as a measure of overall self-regulation. The overall score is calculated by adding together scores on each item. The results can range from 13 to 52 with higher scores indicating better self-regulation. Cronbach’s *α* was 0.76.

Academic performance was measured by asking participants *What grades do you get in school?* They could choose one of the answers: (a) Mostly below Ds, (b) Mostly Ds, (c) About half Cs and half Ds, (d) Mostly Cs, (e) About half Bs and half Cs, (f) Mostly Bs, (g) About half Bs and half As, or (h) Mostly As, based on the survey by the Search Institute (2016). We did not use grade average because there are several subjects (e.g., physical education) in which students have only grades Bs or As and they increase grade average. By asking students which grades they get at school, we tried to get their focus on subjects of higher relevance, which they also have most frequently (Croatian language and mathematics). Scores on this item could range between 1 and 8, where higher scores indicate better academic performance.

Patient Health Questionnaire - PHQ-9 (Kroenke et al., 2001) is a 9-item measure primarily designed to assess symptoms of depression according to DSM-IV criteria and for use in primary health care. Respondents are asked to rate how often they are bothered by each symptom over the past 2 weeks on a scale from 0 (*almost never*) to 3 (*almost every day*). The score is calculated by adding responses to each item. The results can range from 0 to 27, with higher scores indicating more depression symptoms. Cronbach’s *α* was 0.85.

## Results

Table 1 presents descriptive statistics, skewness, and kurtosis for contribution, self-regulation, academic performance, and depression symptoms.

**Table 1:** Descriptive statistics.

	*M*	*SD*	Obtained range	Possible range	Skewness	Kurtosis
Contribution	12.70	3.43	5.00–25.00	0.00–25.00	0.465	0.163
Self-regulation	36.97	6.06	21.00–52.00	13.00–52.00	−0.110	−0.507
Academic performance	5.47	1.55	1.00–8.00	1.00–8.00	−0.430	−0.034
Depression symptoms	9.00	5.68	0.00–27.00	0.00–27.00	0.798	0.271

Variables were screened for outliers and their distributions were analyzed for normality. Inspection of *z*-scores, frequency tables, boxplots, p-p lots, q-q-plots, and histograms did not indicate specific cases that would classify as outliers that needed to be treated. *Z* scores for skewness and kurtosis were significant in some variables, suggesting some deviations from normality. However, these deviations are unproblematic, since large samples as in the present study typically yield significant skewness and kurtosis findings. For the male and female subsample, Levene’s test of homogeneity of variances showed no significant difference among variances. The graphs of *z*-scores of predicted values and errors (*zpred* vs. *zresid*) also did not show deviations from assumption of linearity and homoscedasticity. For the regression analyses, all variance inflation factor (VIF) values were near 1, with the highest value of VIF being 1.082 and the lowest value of tolerance being 0.924, indicating no multicollinearity. This was further confirmed by inspecting eigenvalues, condition indexes, and variance proportions. As the results performed on standardized and unstandardized variables did not differ, we here report the results using the unstandardized variables.

Table 1 also shows that possible and obtained ranges of variables that were measured were very similar. An exception was the obtained minimum for self-regulation, which was higher than possible, as well as the mean for self-regulation, which indicated that results were grouping toward higher values. The mean for depression symptoms was relatively low, which was expected because the sample is non-clinical, but we could see from the obtained range that in this sample we had students who had a maximum score on the PHQ-9 scale. The mean for academic performance (Hedin and Conrad, 1980; Rosenthal et al., 1998) indicated that students mostly got Bs, or partly Bs, and partly Cs in school.

In order to answer our first research question, we used Pearson’s correlation coefficients to examine correlations between variables. Correlations between study variables are presented in Table 2.

**Table 2:** Correlation coefficients.

	Gender^***^	Contribution	Self-regulation	Academic performance
Gender	-			
Contribution	0.13^**^	-		
Self-regulation	−0.03	0.13^*^	-	
Academic performance	0.21^**^	0.10^*^	0.16^**^	-
Depression symptoms	0.24^**^	−0.12^*^	−0.45^**^	−0.16^**^

* *p* < 0.05

** *p* < 0.01

*** Gender was coded such that females got a score of 2 and males 1, so in the following analyses positive correlation with gender would mean that females score higher on the associate variable.

As can be seen from Table 2, contribution was significantly negatively correlated with depression symptoms. It was also positively correlated with self-regulation and academic performance. Correlations were all in the low range. Furthermore, self-regulation had a moderate negative correlation with depression symptoms, while academic performance showed low negative correlation with depression symptoms.

Hierarchical regression analysis was used to examine the effects of contribution on depression symptoms in accordance with our first research question. Also, it was the first step in examining our second research question, that is whether contribution affects depression symptoms through self-regulation and academic performance. Given the consistent gender differences in adolescents’ depression (Weissman and Klerman, 1985; Fanous et al., 2002), as well as a significant correlation between gender and depression symptoms obtained in this study, gender was added in the first step of the hierarchical regression analysis (HRA) to control for its effects, followed by contribution in the second, self-regulation in the third, and academic performance in the fourth step. Gender was scored with 1 for male and 2 for female. Results are shown in Table 3.

**Table 3:** Results of hierarchical regression analysis for depression symptoms as an outcome.

Model	*β*	*R*^2^	Δ *R*^2^	*F*
1.				
Gender	0.25^***^	0.06^*^		25.51^***^
2.				
Gender	27^***^	0.08^**^	0.02^**^	16.72^***^
Contribution	−0.14^**^			
3.				
Gender	0.24^***^	0.27^***^	0.19^***^	47.01^***^
Contribution	−0.07			
Self-regulation	−0.45^***^			
4.				
Gender	0.27^***^	0.29^**^	0.02^**^	38.62^***^
Contribution	−0.06			
Self-regulation	−0.42^***^			
Academic performance	−0.14^**^			

* *p* < 0.05

** *p* < 0.01

*** *p* < 0.001.

As can be seen in Table 3, gender was a significant predictor indicating higher depression symptoms scores in girls. After controlling for the effect of gender, contribution predicted fewer depression symptoms in the second step of the HRA, and after controlling for gender and contribution, self-regulation also predicted lower depression symptoms scores in the third step. Finally, after controlling for the effects of gender, contribution, and self-regulation, academic performance also predicted lower depression symptoms scores. Self-regulation showed the largest effect on depression, while the effects of contribution and academic performance were small. Therefore, with regard to our first research question, contribution was a significant predictor of depression symptoms in adolescents.

**Figure 1:**
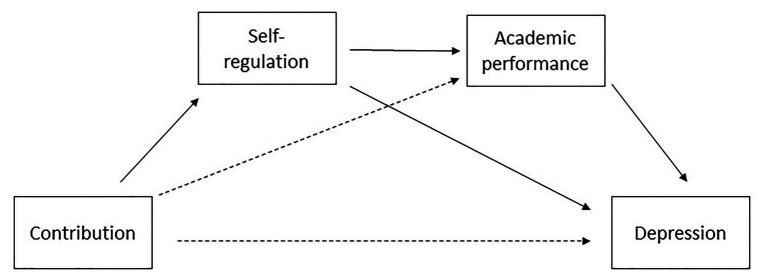
Mediation of contribution on depression symptoms through self-regulation and academic performance with gender as a covariate.

After adding self-regulation in the HRA, the effect of contribution was reduced to a non-significant level suggesting mediation. In order to examine the possible mediation through self-regulation and academic performance, in accordance with our second research question, Hayes’s (2009, 2018) bootstrapping method was used. We opted for Hayes’s PROCESS because it allows for testing multiple mediations instead of testing each mediation separately. Furthermore, PROCESS automatically compares possible paths (through either of the mediators or through both mediators) to examine which is the strongest path. Additionally, PROCESS is an appropriate method for mediation models that rely completely on observed data, especially when there is just one, non-multidimensional independent variable (Hayes et al., 2017), which is the case in our study. Given the significant relationship of gender with contribution, academic performance, and depression symptoms, gender was included in the analysis as a covariate. The summary of mediation analysis is presented in Table 4, and the conceptual diagram is presented in Figure 1.

**Table 4:** Mediation of contribution on depression symptoms through self-regulation and academic performance with gender as a covariate.

**Model summary**						
	*Coefficient*	*SE*	*t*	*p*	**Bootstraping BCa 95% CI**	
					**Lower**	**Upper**
Contribution	−0.243	0.081	−2.979	0.003	−0.4024	−0.0825
Gender	3.061	0.557	5.497	0.000	1.9664	0.4738
**Total effect of contribution on depression**						
	*Effect*	*SE*	*t*	*p*	**Bootstraping BCa 95% CI**	
					**Lower**	**Upper**
	−0.243	0.081	2.979	0.003	−0.4024	−0.0825
**Direct effect of contribution on depression**						
	*Effect*	*SE*	*t*	*p*	**Bootstraping BCa 95% CI**	
					**Lower**	**Upper**
	−0.121	0.072	−1.678	0.094	−0.2637	0.0208
**Indirect effect of contribution on depression**						
			*Effect*	*SE*	**Bootstraping BCa 95% CI**	
					**Lower**	**Upper**
**Total**			−0.121	0.044	−0.2155	−0.0421
1. Self-regulation			−0.099	0.044	−0.1932	−0.0222
2. Self-regulation – academic performance			−0.005	0.003	−0.0161	−0.0012
3. Academic performance			−0.017	0.015	−0.0553	0.0041
**Comparison of indirect effects**						
1–2			−0.094	0.042	−0.1833	−0.0203

The results of the mediation analysis show that contribution predicted self-regulation but not academic performance. However, self-regulation predicted academic performance, which resulted in two significant mediational paths – through self-regulation, and through self-regulation and academic performance. A comparison of two significant mediations indicated that indirect effect through self-regulation and academic performance was stronger than the indirect effect containing only self-regulation. With the inclusion of the two mediators, the effect of contribution to depression symptoms was reduced to an insignificant level, suggesting that the relation was fully mediated by both self-regulation and academic performance. Therefore, with regard to our second research question, our findings suggest that contribution affects depression symptoms through both self-regulation and academic performance.

## Discussion

So far, contribution has mainly been investigated with regard to youth political engagement (Rosenthal et al., 1998) and only recently with regard to positive youth development (Larson, 2000; Lerner et al., 2005, 2006; Jelicic et al., 2007; Lewin-Bizan et al., 2010; Agans et al., 2014; Conway et al., 2015; Wiium, 2017). While recent studies have demonstrated benefits of contribution in terms of psychosocial adjustment (Switzer et al., 1995; Phelps et al., 2007), little is known about its protective effects in terms of adolescents’ mental health. Some studies demonstrated the power of positive youth development (confidence, competence, character, caring, and connection) prospectively, predicting both contribution and depression (Jelicic et al., 2007). There are also studies demonstrating positive effects of adolescents’ volunteering, as well as studies that did not document significant effects (Kirkpatrick Johnson et al., 1998; Phelps et al., 2007; Bowers et al., 2010). We are not aware of any studies that examined the link between contribution and depression, nor the possible mediating factors. Therefore, the purpose of our study was to examine the relationship between contribution and depression symptoms, and a possible mediating role of self-regulation and academic performance in this relationship. We also included gender as a control variable, given the fact that it was significantly positively related with contribution, academic performance, and depression. In addition, previous studies consistently show gender differences in expression of depression symptoms among adolescents (Kuehner, 2003; Galambos et al., 2004). Furthermore, female adolescents exhibit two to three times higher rates of depression than their male peers (Hyde et al., 2008), a finding that seems to be consistent across different cultures (Hopcroft and Burr Bradley, 2007). Similar results are found in samples of Croatian adolescents (Kurtovic and Marcinko, 2011; Vucenovic et al., 2015; Kurtovic, 2020). This was corroborated in our study, as female gender predicted more depression symptoms.

The results of this study supported our assumption that contribution is related to adolescent depression symptoms. They revealed that contribution could predict lower depression symptoms scores in adolescents, which is in line with studies demonstrating that volunteering and community service can have protective effects in terms of adolescents’ well-being (Phelps et al., 2007; Bowers et al., 2010). Behaviors, which include helping people in one’s community (such as helping friends or neighbors, volunteering etc.), are likely to positively affect adolescents’ feeling of self-worth, making them see themselves as good persons, as studies demonstrating positive effects of volunteering and community service on adolescents’ self-esteem suggest (Switzer et al., 1995; Kirkpatrick Johnson et al., 1998). In addition, contribution could lead to more favorable social comparison in adolescents, resulting either from comparing oneself to peers who do not engage in similar activities or from positive feedback from others. Huang (2016) demonstrated positive effects of charitable giving and volunteering on life satisfaction due to more favorable social comparison in adults. However, we are not aware of studies examining the relationship between contribution and social comparison in adolescents, therefore this assumption remains to be examined. Furthermore, behaviors entailed in contribution are proactive in nature and usually give immediate results. As such, they can promote active approach to problems, reduce passivity, as well as promote self-efficacy, and thus protect against depression. We are not aware of any studies examining the effects of contribution on either coping, problem solving, or self-efficacy, therefore this should be examined in future studies. However, self-efficacy is considered to be one of the descriptors of 5Cs of positive youth development, and since contribution is considered to be the 6th C of the positive youth development, it is logical to assume a relation between contribution and self-efficacy (Lerner et al., 2005). There is evidence suggesting that volunteering in adults is related to lower psychological distress (Tabassum et al., 2016) and with slower increase in depressive symptoms (Lum and Lightfoot, 2005). While this certainly can be true for youth, the positive effects of volunteering on stress and symptom moderation in adolescents are yet to be examined. Contribution can also increase a sense of belonging and social connectedness, which can account for its relationship with depression, as some studies suggest (Musick and Wilson, 2003; Creaven et al., 2018). However, studies to date have examined the effects of volunteering on social connectedness in adults, while these effects are not known in adolescents.

Self-regulation also showed to be a potentially significant predictor of lower depression symptoms scores, which is in line with studies demonstrating protective effects of regulatory abilities against psychopathology in childhood and adolescence (Sloan et al., 2017). Emotion regulation, or difficulties with emotion regulation, have been shown as a central factor in development of many psychopathological symptoms and disorders, although cognitive and behavioral regulation are also important, especially as a child moves toward abstract thinking in adolescence (Kring and Sloan, 2009). Specifically, with regard to depression, there are studies demonstrating that emotion regulation has direct links with depression and disorders which are often comorbid, such as anxiety disorders, eating disorders, and borderline personality disorder (Berking et al., 2013; Carpenter and Trull, 2013; Aldao and Dixon-Gordon; 2014). Furthermore, our results are in line with Strauman and Eddington (2017) conceptualization of depression in terms of dysfunctional self-regulation. The inability or difficulty with managing emotions, thoughts and behavior, associated with pursuit of one’s goals, can result in either inability or unwillingness to change strategies in the face of failure, or amend one’s goals if the current one is unattainable, which inevitably leads to the accumulation of failures, and in turn increases the risk for depression. The characteristics of depressed people, which include rigidity in cognition and behavior as well as passiveness in behavior, are in line with this assumption. Furthermore, even without accumulation of failures, fixation on one’s unattainable goals can reduce the likelihood of positive experiences (successes in attaining other goals), which again increases the risk for depression. It is reasonable to assume that both of these processes would be present; therefore, it is not surprising that self-regulation consistently shows effects on depression. In practical terms, this can be applied to virtually every aspect of adolescent functioning: academic success, peer relationships, relationship with parents etc. In academic functioning, inability to adapt to changing external demands (as one progresses through education), to differentiate and adapt learning strategies, regulate one’s perception of own abilities in accordance with feedback and outcomes, and adjust goals, if necessary, can impair not only academic success but also mental health. Inflexibility or inadaptability to others as a response to social cues, difficulty in accepting different perspectives, and insistence on one’s wants and desires can seriously decrease the quality of adolescents’ relationships with both parents and peers, and lead to conflicts and interpersonal stress, all of which increase the risk for depression.

Finally, better academic performance also predicted lower depression symptoms scores in this study, beyond the effect of contribution and self-regulation. Failure in one’s academic aspirations, especially if it is continuous, has been linked to psychopathological symptoms in adolescents (Huang, 2015). While academic performance is often examined as an outcome rather than a causal factor, and there is evidence of internalizing symptoms impairing academic performance (Multon et al., 1991; Malecki and Elliot, 2002), it is justified to examine the effect of academic failure on depression symptoms. Repeated academic failure is stressful and exposure to stress increases the risk for depression (George and Lynch, 2003; McCarty et al., 2008), especially if it is combined with unfavorable interpretations of failure, for example, in terms of personal incompetence or stable and global attributions which lead to helplessness and hopelessness (Metalsky et al., 1993; Flynn and Rudolph, 2007). Furthermore, Cole et al. (2001) argue that failure in important tasks poses a risk for depression due to the development of negative self-schemes. Those self-schemes are then activated throughout different situations and lead to conclusions of personal incompetence, which lead to depression. While sense of competence in one domain can be compensated for by competence in other domains, the risk of developing depression increases if a student feels incompetent in different areas. Furthermore, academic performance is related to other factors, such as motivation, social acceptance, feedback from significant adults, and related rewards, all of which can have an impact on students’ well-being and moderate the effect of academic performance on depression. Herman et al. (2008) found that the effects of low school grades on depression starts around middle childhood and ends in young adulthood, probably because young adults have other domains relevant to their self-perception to compensate for low academic performance. This highlights the impact of academic performance on adolescents’ mental health and the importance of introducing strategies that aim at both improving their academic performance and strengthening their sense of competence in other areas. Promoting adolescents’ involvement in different activities, including contribution, might improve their self-perception even in face of academic failure, by providing other domains for positive self-evaluation.

### Self-Regulation and Academic Performance as Mediators Between Contribution and Depression Symptoms

The second goal of our study was to examine whether self-regulation and academic performance can mediate the relationship between contribution and depression symptoms in adolescents. The reason why we focused on self-regulation as a possible mediator was rooted in findings on the relation between positive youth development and self-regulation within a framework of developmental systems theory (Lerner, 2006). According to the developmental systems theory, mutually beneficial relationships (to the individual and to the context) should result in increased capacity to regulate one’s behavior toward attainment of one’s goals (Gestsdottir and Lerner, 2007). Contribution could be an example of a mutually beneficial relationship between an individual and one’s context and could thus be related to self-regulation. Furthermore, there are studies linking volunteering and community service to better academic performance (Kirkpatrick Johnson et al., 1998; Tangney et al., 2004; Day and Connor, 2016), as well as studies demonstrating the effect of self-regulation on both higher grade point average and better adjustment (i.e., fewer reports of psychopathology, higher self-esteem; Tangney et al., 2004).

The results of the mediation analysis partly support our hypothesis that both self-regulation and academic performance mediate the relationship between contribution and depression symptoms in adolescents. Self-regulation by itself was a significant mediator, which was expected given the fact it had the largest effect on depression symptoms. Prosocial behavior in general, as well as volunteering and community service, have been linked to the ability to delay gratification (Long and Lerner, 1974) and to greater social responsibility and fulfillment of one’s duties. We are not aware of any studies examining the effects of contribution on self-regulation, especially in terms of the development of depression. However, Eisenberg et al. (1997) demonstrated that emotionality and emotion regulation in competent social behavior are associated with empathy for other people’s problems and are one of the most important indicators of competent socio-emotional functioning. As a consequence of that, contribution should promote the development of empathy for other people which can improve adolescents’ socio-emotional functioning, thus protecting them from emotional problems. Furthermore, the purpose of self-regulation is not only about attaining one’s goals, but also about consolidating one’s behavior with external expectations, usually from significant others and social norms. Thus, contribution can also affect adolescents’ ability to regulate their behavior to conform to socially desirable behaviors. This seems to be especially important in adolescence, given that their executive functions are still developing. A central aspect of self-regulation are executive functions, including planning, inhibition, self-control, and correcting errors among others, which are important determinants of adaptive behavior (Miyake et al., 2000; Lewis and Todd, 2007). We can assume that adolescents involved in community work and other prosocial activities should be able to plan and regulate various obligations and activities such as school, community work, and sports. Being exposed to role models and gratification associated with contribution may facilitate the development of executive functions.

Finally, academic performance alone was not a significant mediator, which is neither in line with studies suggesting positive effects of prosocial behavior on academic achievement (Caprara et al., 2000; Carpenter and Trull, 2013) nor with our assumption that contribution would affect depression symptoms because it affects academic performance. However, it was in line with studies that used the same measures of contribution and academic performance (Wiium, 2017). Nevertheless, even though academic performance by itself did not show to be a significant mediator, the path containing self-regulation and academic performance was significantly stronger than the one containing only self-regulation. Therefore, our results suggest that contribution may protect against depression symptoms because it promotes self-regulation, which in turn can lead to better academic performance, and fewer depression symptoms. As previously mentioned, self-regulation is related to better academic performance (Tangney et al., 2004; Day and Connor, 2016), presumably because it enables self-generated thoughts and behaviors that are systematically oriented toward the attainment of learning goals (Schunk and Zimmerman, 2003). Moreover, self-regulation enables a person to postpone immediate reinforcement (e.g., a pleasurable activity) in favor of a different and usually greater future reinforcer (Rachlin, 1992), for instance, a better academic performance.

Our results are in line with Strauman and Eddington (2017) notion of depression as a result of self-regulatory failure. According to Strauman and Eddington (2017), self-regulation is principal in determining the way people approach situations that may result in them gaining something and situations in which they stand to lose something desirable. They propose that in depression, deficient self-regulation affects the way adolescents approach challenges with regard to academic performance. Strauman and Eddington (2017) also propose that self-regulation is a proximal factor which explains the influence of more distal factors (such as temperament, socialization, physical, and social environment) on a person’s functioning (emotions, motivation, behavior, and cognition). Translated into the present study, self-regulation could explain the influence of contribution (as a result of socialization and environment) on goal pursuit (academic performance), which then can affect depression symptoms.

In conclusion, our results suggest that contribution can be a protective factor against depression in adolescents. Although the effect was small, it should not be neglected. Depression is a multi-determined disorder and compared to more salient factors, such as self-esteem, the effect of contribution is expectedly small. Nevertheless, a meaningful path through self-regulation and academic performance suggests that engaging in helping behaviors can have far-reaching positive effects in terms of facilitating the development of useful skills and competences. The promotion of benefits of contribution should not be focused only on youth but should be considered as a one way of preventing the development of or depleting depression symptoms in all age groups. Also, contribution is mutually beneficial, to the individual as well as the community, promoting values such as volunteering, helping one another and giving back to one’s community.

## Limitation and Implications

The study has some limitations. We used self-report questionnaires as measures of self-regulation, depression symptoms, academic performance, and contribution. Students could give socially desirable responses, although we try to prevent that by informing students that their participation in the study is voluntary and anonymous. In addition, the correlational nature of the study does not allow drawing conclusions about causality. That is, does contribution cause changes in self-regulation and academic performance, leading to reduced depression symptoms or do less depressed individuals engage more often in contribution and have higher self-regulation and academic performance? In this respect, it is important to mention that there are studies demonstrating reciprocal effects of well-being on involvement in community activities (Thoits and Hewitt, 2001), as well as studies demonstrating that students with higher academic achievement are more likely to volunteer (Raskoff and Sundeen, 1994). However, reciprocal relations between community involvement and different indicators of well-being (such as depression symptoms or academic performance) are expected because of simultaneous effects of social causation and social selection (Thoits and Hewitt, 2001). That is, while adolescents with better well-being and better academic performance may be more inclined to engage in community service due to already developed sets of personal characteristic, goals, or due to parental influence, that involvement may provide them with further benefits. Likewise, as our results suggest, adolescents who have not had experience with contribution, as well as adolescents at risk, may benefit from it.

Results of our and similar studies have some important implications for treatment and prevention of mental health problems in adolescents, as well as promotion of positive youth development, especially taking into consideration Croatian circumstances. Croatia is a transitional society, which means that Croatian adolescents are passing the transformation from childhood to adulthood while the whole society is experiencing major changes. Financial, social, and political consequences of the Croatian war for independence are still present; also, Croatia was affected by the economic crisis that had negative effects on young people and their families who were largely impoverished (Ilisin and Spajic Vrkas, 2015). In such an environment where the focus of society is on raising the economic standard, there may be a lack of social support for young people who are then at greater risk for developing risk behaviors and mental health problems. Knowledge about positive effects of engagement in helping activities can help clinicians tailor interventions for at risk adolescents, especially within group therapy, which include active participation in helping activities, as well as advising parents and teachers to design structured, helping activities for adolescent to engage in, which might help alleviate their symptoms. Contribution seems to be beneficial for one’s well-being for three reasons: (1) the positive experience of helping others or one’s community can serve as a validation of one’s self-worth, (2) adolescents are likely to gain validation from others, adults, and peers alike, that facilitates positive relationships, and (3) it is likely that it benefits their self-regulatory skills. However, if adolescents are going to benefit from these findings, contribution activities need to be valued in one’s community, they need to be structured, preferably supervised by adults, and they should be relatively accessible, ideally available through school or local community (Lerner, 2004). We are not aware of any such programs on an institutional level, nor programs on a local community level, which are widely available. Adolescents do have opportunities for contribution within programs of certain NGOs, but they are anecdotal and include a small number of volunteers. Schools are an ideal venue for implementing such activities, given that adolescents spend a large proportion of their time in school, form important relationships there, and it is a place where many of their goals can be pursued. Furthermore, our results imply that self-regulation and academic performance could have an important role in explaining the effects of contribution on depression symptoms in adolescents. Educational systems, like Croatian, are mostly oriented toward the development of cognitive competencies and attaining academic goals, while the development of emotional and social competencies and skills is not as valued (Dautovic and Dedic Bukvic, 2020). Taking into consideration the importance of noncognitive factors, our findings emphasize the importance of promoting contribution and positive youth development in schools.

## Data Availability Statement

The datasets presented in this study can be found in online repositories. The names of the repository/repositories and accession number(s) can be found at: University of Bergen, PYD cross-national project archive, file pyd_croatia_2018_datafile_v2.sav, link: https://universityofbergen.sharepoint.com/sites/TEAM_PYDCrossNational_Project/Shared%20Documents/General/PYD%20Database/Croatia/pyd_croatia_2018_datafile_v2.sav.

## Ethics Statement

The studies involving human participants were reviewed and approved by Ethics Committee of the Faculty of Humanities and Social Sciences in Osijek (class: 602-04/18-01/29, number: 2158-83-02-18-2) and NSD - Norwegian Centre for Research Data (approval number is 51708/3/IJJ). Written informed consent from the participants’ legal guardian/next of kin was not required to participate in this study in accordance with the national legislation and the institutional requirements.

## Author Contributions

GV organized the database. AK wrote the first draft of the manuscript. All authors contributed to conception and design of the study, performed the statistical analyses, wrote sections of the manuscript, contributed to manuscript revision, read, and approved the submitted version.

### Conflict of Interest

The authors declare that the research was conducted in the absence of any commercial or financial relationships that could be construed as a potential conflict of interest.
